# Large-Vessel Giant Cell Arteritis following COVID-19—What Can HLA Typing Reveal?

**DOI:** 10.3390/diagnostics13030484

**Published:** 2023-01-28

**Authors:** Maja Stojanovic, Aleksandra Barac, Ana Petkovic, Nikola Vojvodic, Strahinja Odalovic, Zorana Andric, Rada Miskovic, Dragana Jovanovic, Sanja Dimic-Janjic, Sanja Dragasevic, Sanvila Raskovic, Mihailo I. Stjepanovic

**Affiliations:** 1 Clinic for Allergy and Immunology, University Clinical Center of Serbia, 11000 Belgrade, Serbia; 2 Faculty of Medicine, University of Belgrade, 11000 Belgrade, Serbia; 3 Clinic for Infectious and Tropical Diseases, University Clinical Center of Serbia, 11000 Belgrade, Serbia; 4 Department of Radiology, Center of Stereotaxic Radiosurgery, Clinic of Neurosurgery, University Clinical Center of Serbia, 11000 Belgrade, Serbia; 5 Clinic for Neurology, University Clinical Center of Serbia, 11000 Belgrade, Serbia; 6 Center for Nuclear Medicine and PET, University Clinical Center of Serbia, 11000 Belgrade, Serbia; 7 Tissue Typing Department, Blood Transfusion Institute of Serbia, 11000 Belgrade, Serbia; 8 Clinic for Pulmonology, University Clinical Center of Serbia, 11000 Belgrade, Serbia; 9 Clinic for Gastroenterohepatology, University Clinical Center of Serbia, 11000 Belgrade, Serbia

**Keywords:** COVID-19, SARS-CoV-2 infection, large-vessel vasculitis, giant cell arteritis, HLA, autoimmunity

## Abstract

Giant cell arteritis (GCA) is an immune-mediated vasculitis that affects large arteries. It has been hypothesized that viruses may trigger inflammation within the vessel walls. Genetic studies on human leukocyte antigens (HLAs) have previously reported HLA-DRB1*04 as a susceptible allele for GCA and HLA-DRB1*15 as a protective allele for GCA. Here, we discuss the clinical presentation, laboratory findings, HLA class I and class II analysis results, and management of patients with extracranial large-vessel (LV) GCA, detected at least six weeks after recovery from COVID-19. This case series encompassed three patients with LV-GCA (two males and a female with an age range of 63–69 years) whose leading clinical presentation included the presence of constitutional symptoms and significantly elevated inflammatory markers. The diagnosis of LV-GCA was confirmed by CT angiography and FDG-PET/CT, revealing inflammation in the large vessels. All were treated with corticosteroids, while two received adjunctive therapy. By analyzing HLA profiles, we found no presence of the susceptible HLA-DRB1*04 allele, while the HLA-DRB1*15 allele was detected in two patients. In conclusion, LV-GCA may be triggered by COVID-19. We highlight the importance of the early identification of LV-GCA following SARS-CoV-2 infection, which may be delayed due to the overlapping clinical features of GCA and COVID-19. The prompt initiation of therapy is necessary in order to avoid severe vascular complications. Future studies will better define the role of specific HLA alleles in patients who developed GCA following COVID-19.

## 1. Introduction

Coronavirus disease 2019 (COVID-19), caused by severe acute respiratory syndrome coronavirus 2 (SARS-CoV-2), is a new illness that is predominantly manifested with respiratory symptoms [1]. Various immunological phenomena have been observed during the course of the disease, which may lead to rheumatologic illnesses following the resolution of an acute infection [2,3]. Large-vessel vasculitis (LVV) was demonstrated on positron emission tomography combined with low-resolution computed tomography using fluorodeoxyglucose (FDG-PET/CT) in a substantial proportion of patients with persistent symptoms following recovery from COVID-19 [4]. Similarly, the development of GCA (with different clinical presentations) after full recovery from COVID-19 has been reported in multiple cases [5,6,7,8,9].

Giant cell arteritis (GCA) is considered a polygenic disease of unknown etiology, occurring mainly in individuals over the age of 50 years [10]. Although GCA is not a truly infectious vasculitis, it is proposed that an infection may trigger inflammation within the vessel walls [10]. Advances in non-invasive arterial imaging have improved the understanding of the frequency of large-vessel (LV) GCA, predominantly affecting the aorta and its major branches, demonstrated in up to 83% of patients within the overlapping phenotypes, including classic cranial arteritis and extracranial LV-GCA [11]. Genetic studies have reported various disease associations with major histocompatibility complex (MHC)-encoding human leukocyte antigens (HLAs) [12,13]. The presence of HLA-DRB1*04:01 and HLA-B*15:01 increases the susceptibility to cranial and extracranial GCA [14,15]. A recent meta-analysis revealed the protective effects of HLA-DRB1*15, HLA-DRB1*16, and possibly HLA-DRB1*01 alleles [15]. Japanese authors have identified the susceptible HLA-DR4 allele (accounting for the HLA-DRB1*04 and HLA-DRB1*01 alleles) in a patient who developed LVV associated with SARS-Cov-2 infection [5].

Here, we discuss the clinical presentation, laboratory findings, HLA testing results, and management of three patients with extracranial LV-GCA following SARS-CoV-2 infection. To the best of our knowledge, this is the first study to report a comprehensive analysis of both the HLA class I and class II profiles in patients with LV-GCA associated with COVID-19.

## 2. Materials and Methods

We aimed to present a case series of three patients and a relevant literature review regarding the association of LVV, GCA, and COVID-19.

### Search Strategy

We used the PubMed and Medline databases and searched for relevant literature published until the end of October 2022 using the keywords “COVID-19” and/or “SARS CoV-2” and “large vessel vasculitis” and “COVID-19” and/or “SARS CoV-2” and “giant cell arteritis”. The articles written in English were reviewed. After the initial selection of articles, we reviewed the bibliographies of the obtained articles that search for additional references not identified by the previous search. After excluding the irrelevant articles relating to patients with large-vessel vasculitis developed at a young age, pediatric cases, vasculitis in relation to the SARS-CoV-2 vaccine, and infective aortitis, we identified six articles which were included in the review.

## 3. Results

### 3.1. Presentation of Cases

#### 3.1.1. Patient 1

**Figure 1:**
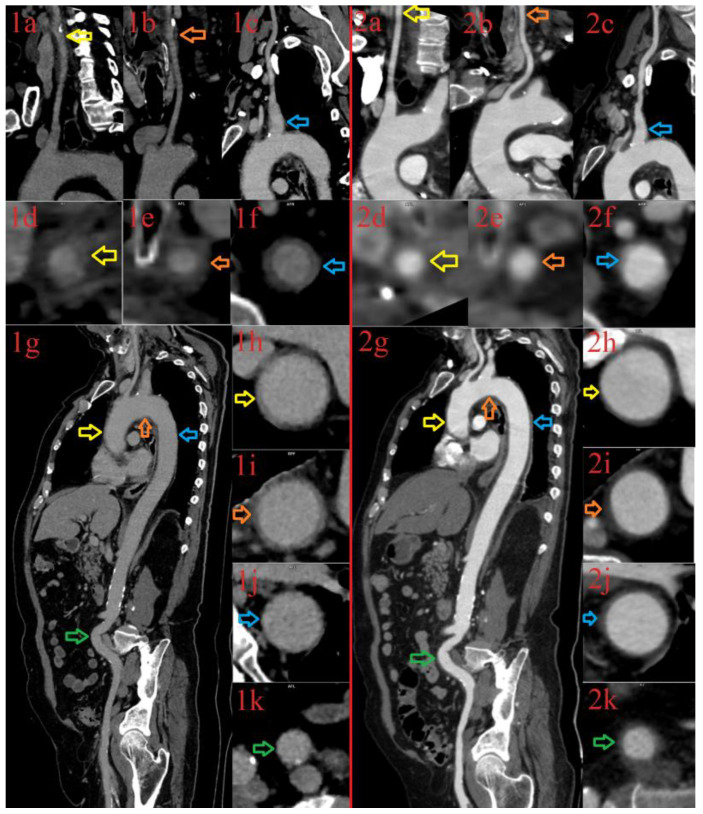
CT aortography of a 69-year-old male with large-vessel vasculitis performed at baseline (Images (**1a**–**1k**)) and at the six months follow-up examination (Images (**2a**–**2k**)). The baseline CT depicts wall thickening of the aortic arch branches that is barely seen after six months in the right carotid artery (Baseline: Image (**1a**,**1d**); Follow-up: Image (**2a**,**2d**); yellow arrows), the left carotid artery (Baseline: Image (**1b**,**1e**), Follow-up: Image (**2b**,**2e**); orange arrows), and the left subclavian artery (Baseline:: Image (**1c**,**1f**); Follow-up: Image (**2c**,**2f**); blue arrows). In addition, the baseline CT aortography depicts the inflammation of the ascending aorta (Image (**1g**,**1h**), yellow arrows) and the aortic arch (Image (**1g**,**1i**), orange arrows), which was significantly thinner after six months (The ascending aorta: Image (**2g**,**2h**), yellow arrows; The aortic arch: Image (**2g**,**2i**), orange arrows). On the contrary, the descending thoracic aorta (Image (**1g**,**1j**), blue arrows) and iliac arteries (Image (**1g**,**1k**), green arrows) are intact at baseline CT, and appeared inflamed after six months (The descending thoracic aorta: Image (**2g**,**2j**), blue arrows; Iliac arteries: Image (**2g**,**2k**), green arrows).

We previously published our first case of a 69-year-old male who developed LV-GCA and presented with symptoms of systemic and cerebral vasculitis six weeks after recovery from a mild form of COVID-19. The patient was treated with pulsed corticosteroid therapy, therapeutic plasma exchange (TPE), and tocilizumab. Following a significant improvement in clinical and laboratory parameters, we concluded that the therapy led to a complete recovery within 2 months after its initiation [8]. However, we continued to follow the patient, who eventually experienced a laboratory exacerbation of LV-GCA (erythrocyte sedimentation rate (ESR) 100 mm/h and C-reactive protein (CRP) 151.7 mg/L) six months later. Additional immunological testing revealed a lower serum immunoglobulin (Ig) G concentration (4.4 g/L) within a low total gamma fraction in protein electrophoresis (9.4% of total proteins). The immunophenotyping of peripheral blood lymphocytes showed a significant decrease in the following: total lymphocytes (ly)—743/μL (normal range 1140–3380/μL), CD3^+^T-ly—566/μL (780–2240), helper CD3^+^CD4^+^T-ly—386/μL (490–1640); cytotoxic CD3^+^CD8^+^ T-ly—150/μL (170–880), natural killer (NK) CD3^+^CD16^+^CD56^+^cells—50/μL (80–490), and a normal count of CD19^+^ B-ly—90/μL (80–490). A significant lymphopenia affecting CD4^+^ and CD8^+^ T-ly and NK cells as well as hypogammaglobulinemia was considered as a consequence of the applied treatment (TPE, immunosuppression). In the presence of an obvious inflammatory relapse, before the introduction of an additional immunosuppressive drug, we repeated the computed tomography angiography (CTA) of the aorta and the branches. A CTA was performed six months after the initial imaging showed an interesting picture of a migratory arteritis, which was consistent with the initial diagnosis of LV-GCA (Figure 1).

The patient was given a single dose of intravenous immunoglobulin (0.5 g/kg of BW), and azathioprine was introduced (2 mg/kg of BW). The treatment was continued with a low dose of prednisone and azathioprine, leading to a complete laboratory improvement in the inflammatory markers and IgG concentrations during the last follow-up in September 2022. Additional testing included the typing of HLA class I and class II, revealing the presence of HLA A*2 and A*24, B*51 and B*57, DRB1*15 and DRB1*16, and DQB1*05 and DQB1*06 allelic groups.

#### 3.1.2. Patient 2

The second case was a 63-year-old female who was diagnosed with COVID-19 in October 2021 after she presented with low-grade fever and fatigue, as well as slightly elevated CRP. SARS-CoV-2 infection was confirmed by a RT-PCR test. She did not receive any specific treatment. She was not vaccinated against SARS-CoV-2. Her medical history revealed that she suffered from hypertension and type II diabetes, but both conditions were well controlled with appropriate oral treatments.

**Figure 2:**
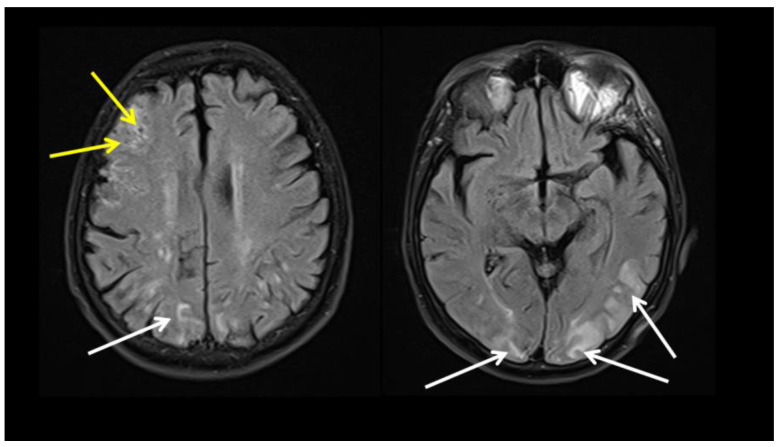
Brain MRI (FLAIR sequence) shows a combination of inflammatory lesions with involvement of U-fibers (white arrows) and cortical and subcortical vascular lesions with microbleeding (yellow arrows), typical for small vessel vasculitis.

Four weeks later, in November 2021, the patient experienced an acute confused state and head version to the left with bilateral motor manifestation upon arrival at the emergency unit. A brain MRI showed multiple hyperintense supratentorial lesions, typical for small vessel vasculitis (Figure 2), while an electroencephalogram showed a mild slowing of background activity. A thorough neurological work-up did not reveal any significant findings. Antinuclear antibodies, anti-cardiolipin IgG and IgM, and anti-neutrophil cytoplasmic antibodies were all negative, and serum immunoglobulins were within the normal range. A diagnosis of COVID-19-associated encephalitis was made, and 40 mg of prednisone was given daily, as well as other supportive therapy, leading to significant clinical improvement.

**Figure 3:**
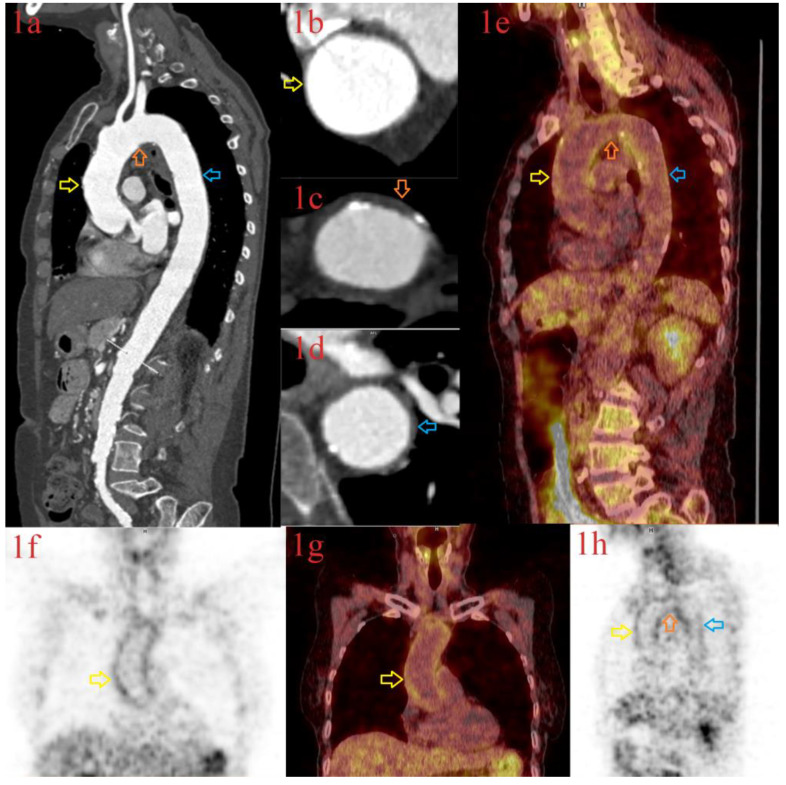
CT aortography (Images (**1a**–**1d**)) and Whole-body FDG PET-CT (Images (**1e**–**1h**)) in a 63-year-old female with large-vessel vasculitis showing inflammation of the aortic wall at the corresponding levels on the following localization: the ascending aorta (CT: Image (**1a**,**1b**); PET-CT: Image (**1e**–**1h**); red arrows), the aortic arch (CT: Image (**1a**,**1c**); PET-CT: Image (**1e**,**1h**); orange arrows) and the descending thoracic aorta (CT: Image (**1a**,**1d**); PET-CT: Image (**1e**,**1h**); blue arrows).

Due to the reappearance of constitutional symptoms (low-grade fever and malaise) and elevated CRP (18.3 mg/L) two weeks later, the CTA of the entire aorta and the branches was performed. The walls of the ascending aorta and the aortic arch appeared thickened (Figure 3, Images 1a–1d). The diagnosis of LV-GCA was confirmed by performing a whole-body FDG-PET/CT, showing the inflammation of the ascending aorta, the aortic arch, and the thoracic and abdominal aorta (Figure 3; Images 1e–1h). The corticosteroid treatment was continued and gradually tapered. Almost a year later, during the last visit, in September 2022, no detectable signs of clinical or laboratory relapses were found, with the continuation of a 5 mg daily dose of prednisone. Genetic testing included the typing of HLA class I and class II, revealing the presence of HLA A*02 and A*11, B*27 and B*35, DRB1*14 and DRB1*15, and DQB1*05 and DQB1*06 allelic groups.

#### 3.1.3. Patient 3

The third case is a 68-year-old male, hospitalized at the Clinic of Infectious and Tropical diseases in June 2022 due to persistent low-grade fever, malaise, a loss of appetite, and a weight loss of 8 kg during the last month. Significantly elevated acute phase reactants (70 mm/h of ESR, 53.9 mg/L of CRP, and 7.2 g/L of fibrinogen) were measured. Previous medical history revealed a mild RT-PCR-confirmed case of COVID-19, two months before manifesting with a loss of smell, a fever, and a cough, but without confirmed lung infection following a chest X-ray. All laboratory parameters were within the normal range. No treatment was administered. He was fully immunized against SARS-CoV-2, and the last of three doses of the Sinopharm inactivated vaccine (Vero cells) was given four months before. According to the medical history, he had stable arterial hypertension.

**Figure 4:**
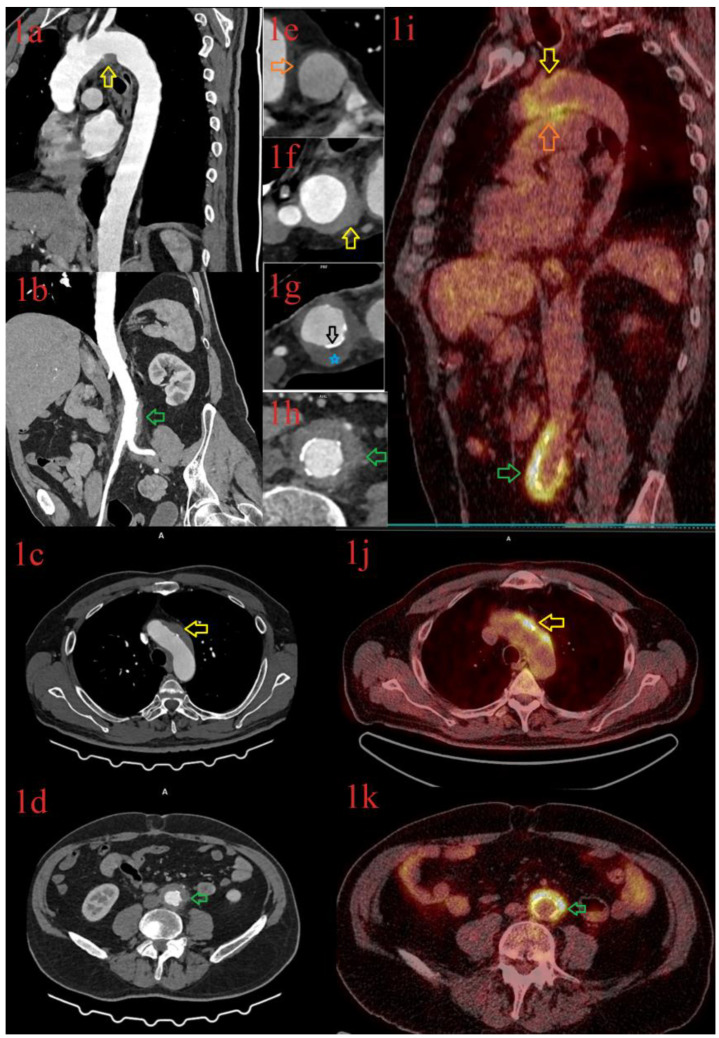
CT angiography (Images (**1a**–**1h**)) and Whole-body FDG PET-CT (Images (**1i**–**1k**)) in a 68-year-old male patient with large-vessel vasculitis showing vessel inflammation with asymmetric hypodense wall thickening (Image (**1g**), blue star) and intimal calcification (Image (**1g**), black arrow) and avid FDG uptake at the same level on the following localizations: the aortic arch (CT: Image (**1a**,**1c**,**1f**); PET-CT: Image (**1i**) I (**1j**); yellow arrows), pulmonary trunk (CT: Image (**1e**), PET-CT: Image (**1i**); orange arrows), and the abdominal aorta (CT: Image (**1b**,**1d**,**1h**); PET-CT: Image (**1i**) I (**1k**), green arrows).

After performing an extensive diagnostic work-up, an acute infection, as a cause of the presenting symptoms, was excluded. A CTA revealed the wall thickening of the thoracic aorta with intimal calcifications. Thickening was seen in the infrarenal segment of the abdominal aortic wall, which was surrounded by an inflammatory muff with the typical morphological features of periaortitis (Figure 4, Images 1a–1h). The patient was diagnosed with LV-GCA and daily treatment of 40 mg of prednisone was started, leading to temporary clinical and laboratory improvements. Four months later, however, while tapering the dose of prednisone to 20 mg, the patient appeared to have constitutional symptoms with high ESR (90 mm/h), CRP (24 mg/L), and fibrinogen (8.9 g/L) concentrations. FDG-PET/CT revealed an intense inflammation of the right subclavian artery, ascending aorta, pulmonary arteries, aortic arch, and abdominal aorta (Figure 4, Images 1i–1k). Azathioprine, along with a daily 30 mg dose of prednisone, was given. Two months later, in November 2022, the inflammatory parameters were significantly improved, and we continued to taper the dose of prednisone. Genetic testing revealed the presence of HLA A*11 and A*30, B*13 and B*40, DRB1*07 and DRB1*11, and DQB1*02 and DQB1*03 allelic groups.

## 4. Discussion

COVID-19 is characterized by diverse clinical presentations, ranging from asymptomatic infection to mild flu-like symptoms, or even fatal respiratory failure and multiorgan dysfunction [16]. In addition, many patients have experienced prolonged symptoms persisting weeks to months after the initial disease onset with occasionally varied longer-term sequelae [17]. Hence, it is not surprising that there is an important need to question which factors determine the mechanisms driving an immune response to SARS-CoV-2 to be helpful or harmful for a particular individual.

The pathogenesis of GCA is far from being completely understood. It is currently accepted that GCA is an antigen-driven autoimmune disease in which pathogenesis, including both innate and adaptive immune responses, has a direct contribution [18]. Interestingly, Varga and colleagues have demonstrated the direct infection of endothelial cells by SARS CoV-2, with a diffuse endothelial inflammation followed by endothelitis, apoptosis, and mononuclear cell infiltrations within the vascular intima [19]. Based on these data, the virus can invade vessel walls and cause vasculitis.

In June 2020, a group of Japanese authors reported the first case of a 71-year-old man who developed LVV associated with SARS CoV-2 infection [5]. Until November 2022, only a few case reports (summarized in Table 1), mainly in the form of short letters, have been reported. Of particular note is the observation that clinicians have faced significant diagnostic and clinical challenges due to the masquerading symptoms of SARS CoV-2 infection to GCA during the COVID-19 pandemic, including constitution symptoms, elevated CRP, fever, headache, and myalgia [20]. Shared and distinct features of GCA and COVID-19 have been comprehensively summarized and discussed in a recent review published by Mehta and colleagues [21].

Previous studies have shown that GCA is a heterogeneous syndrome with different clinical phenotypes mainly correlating with the dominant patterns of immune response [23]. It comprises overlapping phenotypes including the classic cranial forms of arteritis [c-GCA] and extra-cranial GCA, otherwise termed LV-GCA [24]. For a long time, the 1990 American College of Rheumatology (ACR) classification criteria have been used to make an easier discrimination between different vasculitides [25]. However, newly designed ACR/EULAR classification criteria for GCA have included an assessment of more clinical symptoms and specific imaging procedures, such as FDG-PET/CT [26]. Advancements in imaging techniques, such as Doppler ultrasound, nuclear magnetic resonance angiography, CTA, and/or FDG-PET/CT, have led to improvements in the diagnosis of LV-GCA [11]. Accordingly, the diagnosis of LV-GCA in all of our cases was confirmed by CTA and FDG-PET/CT, revealing the inflammation of the large-vessel walls.

According to the recent recommendations, a clinically suspected diagnosis of LV-GCA may be confirmed by imaging (CTA, FDG-PET/CT) without a need for any additional testing, such as temporal artery biopsy (TAB), and treatment with a high dose of corticosteroids (the equivalent of a daily 40–60 mg dose of prednisone) should be initiated immediately for the induction of remission. Adjunctive therapy, along with disease-modifying drugs (DMARDs), should be used in selected patients with a refractory or relapsing GCA [27,28]. There was only one patient who developed a feature of cranial GCA (c-GCA). Except for a headache reported by this patient, no other patients appeared with typical symptoms of c-GCA, such as jaw or tongue claudication, scalp tenderness, or visual impairment. A temporal artery abnormality was detected in the first patient on a brain MRI. Due to the overlapping features of GCA and COVID-19 (including headache, fever, elevated CRP, and cough), the presence of jaw claudication and visual impairment seem to be more discriminatory [21].

Surprisingly, some of the previously reported cases appeared to be a spontaneously resolved form of GCA [5,22]. This observation has created some dilemmas about the various difficulties in discriminating between COVID-19-associated autoimmune vasculitis and LVV with spontaneous resolution, which are more suggestive of an infective aortitis [29]. Moreover, Dhakal and colleagues described a case of inflammation of the infrarenal aortic wall, thought to be a case of aortitis that is secondary to COVID-19 infection, with a gradual complete resolution on an oral daily 60 mg dose of prednisone on day 9 [30]. In contrast to these cases of aortic inflammation with a spontaneous resolution and no anti-inflammatory treatment, mainly detected concomitantly with COVID-19, all of our patients experienced symptoms after full recovery and at least six weeks apart from COVID-19 detection. Sollini et al. have reported the largest cohort of LVV detected using FDG-PET/CT in patients with persistent symptoms after COVID-19; however, details regarding the clinical presentation, clinical course, and management of patients have not been provided [4].

There is accumulating evidence that supports the role of genetic factors in developing GCA. The gene from the HLA locus that has commonly been associated with GCA in Caucasians is HLA DRB1*04. For a long time, it was considered the only genetic association with GCA that has been replicated in independent cohorts [31]. Carriers of this gene were considered to be at a higher risk of developing GCA and for suffering complications such as visual loss, and may also be at higher risk of developing resistance to corticosteroids [32,33,34,35]. With this in mind, and having previously identified the presence of the susceptible DRB1*04 allele in the patient with the LVV associated with COVID-19 [5], we analyzed the HLA class I and class II alleles in our patients. None of the patients carried the susceptible DRB1*04 allele. In contrast, we identified the presence of the HLA-DRB1*15 allele, previously identified as an allele with a putative protective effect, while one of the analyzed patients was a carrier of two (DRB1*15 and DRB1*16) protective alleles, according to the recent meta-analysis [15]. However, a better understanding of the capacity of microorganisms to trigger autoreactive T-cells in patients with GCA may reveal the new pathogenetic roles of the specific HLA alleles, similar to the data obtained from immunogenetic analysis in rheumatoid arthritis [36].

To sum up, we hypothesized that COVID-19 triggered the onset of LV-GCA in our patients. It may be possible that viral peptides, in the context of a specific HLA class II allele, are able to trigger the activation of antigen-specific (autoreactive) CD4+ T-cells, leading to autoimmune-mediated inflammation within the vessel wall. Further studies may clarify the exact mechanisms of the activation of SARS-Cov-2-triggered autoreactive T-cells.

## 5. Conclusions

LV-GCA may be considered an autoimmune disease triggered by SARS-CoV-2 infection, one of the broad spectra of manifestations within post-COVID-19 syndrome. However, many overlapping features of GCA and COVID-19 may lead to the delayed recognition of LV-GCA, indicating the need to highlight the potential link between SARS-CoV2 and the development of LV-GCA. The prompt initiation of therapy is necessary in order to avoid severe vascular complications. Future studies will better define the pathogenetic roles of specific HLA alleles in patients who developed GCA following SARS CoV-2 infection.

## Figures and Tables

**Table 1:** Cases of LVV associated with COVID-19: clinical, laboratory features and management.

Author	Age/Sex	Clinical Presentation, Laboratory Findings	Timing of Symptoms after COVID-19	Diagnosis	Treatment	Testing	Outcome
Oda R, et al., 2020 [5]	73 years, male	Persistent spiking fever, elevated ESR and CRP	concomitantly	LV-GCA	NSAID	CTA, FDG PET-CT	Recovered without treatment
Riera-Marti N, et al., 2021 [22]	50 years, male	High fever, headache, temporal artery thickening, TM joint pain	concomitantly	c/LV-GCA	No treatment	Doppler US FDG PET-CT	Recovered without treatment
Jonathan G, et al., 2021 [7]	47 years, male	PAAM, headache, jaw claudication	8 weeks	PAMM TAB–negative GCA	High dose CS Tocilizumab	Ophthalmic diagnostic tests, TAB	Improvement on treatment
Ivanovic J, et al., 2022 * [8]	69 years, male	Headache, fever, elevated ESR, CRP, Fib, IL-6	6 weeks	LV-GCA Cerebral vasculitis	High dose CS, TPE Tocilizumab, IVIG, AZA	FDG PET CT Brain MRI	Improvement on treatment
Aryal B, et al., 2022 [20]	72 years, female	Headache, blurred vision, transient vision loss, abdominal pain	8 weeks	TAB-negative GCA	High dose CS, AZA, MTX	Clinical presentation Laboratory tests	Improvement on treatment
Szydełko-Paśko U, et al., 2022 [6]	69 years, female	Vision loss [left eye] Headache	2.5 weeks	AAION c-GCA	High dose CS, MTX	Ophthalmic diagnostic tests	Improvement In treatment

ESR: erythrocyte sedimentation rate; CRP: C-reactive protein; LV: Large vessel; c: cranial; GCA: giant-cell arteritis; NSAID: non steroid anti-inflammatory drug; CTA: computed tomography angiography; FDG-PET/CT: positron emission tomography with a low-resolution computed tomography using fluorodeoxyglucose: TM: temporo-mandibular; PAMM: paracentral acute middle maculopathy; TPE: therapeutic plasma exchange; MRI: magnetic resonance imaging; IVIG: intravenous immunoglobulins; CS: corticosteroids; MTX: methotrexate; AZA: azathioprine; AAION: arteritic anterior ischemic optic neuropathy; * follow-up data on this case have been reported in this article (Patient 1 in this paper).

## Data Availability

The data presented in this study are available upon reasonable request from the corresponding author.
